# Mortality in Cancer Patients With COVID-19 Who Are Admitted to an ICU or Who Have Severe COVID-19: A Systematic Review and Meta-Analysis

**DOI:** 10.1200/GO.21.00072

**Published:** 2021-08-18

**Authors:** Amogh Rajeev Nadkarni, Swapna C. Vijayakumaran, Sudeep Gupta, Jigeeshu V. Divatia

**Affiliations:** ^1^ Department of Anaesthesiology, Critical Care and Pain, Tata Memorial Hospital, Homi Bhabha National Institute, Mumbai, India; ^2^ Department of Medical Oncology, Tata Memorial Centre, Homi Bhabha National Institute, Mumbai, India

## Abstract

There are scarce data to aid in prognostication of the outcome of critically ill cancer patients with COVID-19. In this systematic review and meta-analysis, we investigated the mortality of critically ill cancer patients with COVID-19.

**METHODS:**

We searched online databases and manually searched for studies in English that reported on outcomes of adult cancer patients with COVID-19 admitted to an intensive care unit (ICU) or those with severe COVID-19 between December 2019 and October 2020. Risk of bias was assessed by the Modified Newcastle-Ottawa Scale. The primary outcome was all-cause mortality. We also determined the odds of death for cancer patients versus noncancer patients, as also outcomes by cancer subtypes, presence of recent anticancer therapy, and presence of one or more comorbidities. Random-effects modeling was used.

**RESULTS:**

In 28 studies (1,276 patients), pooled mortality in cancer patients with COVID-19 admitted to an ICU was 60.2% (95% CI, 53.6 to 6.7; I^2^ = 80.27%), with four studies (7,259 patients) showing higher odds of dying in cancer versus noncancer patients (odds ratio 1.924; 95% CI, 1.596 to 2.320). In four studies (106 patients) of patients with cancer and severe COVID-19, pooled mortality was 59.4% (95% CI, –39.4 to 77.5; I^2^ = 72.28%); in one study, presence of hematologic malignancy was associated with significantly higher mortality compared with nonhematologic cancers (odds ratio 1.878; 95% CI, 1.171 to 3.012). Risk of bias was low.

**CONCLUSION:**

Most studies were reported before the results of trials suggesting the benefit of dexamethasone and tocilizumab, potentially overestimating mortality. The observed mortality of 60% in cancer patients with COVID-19 admitted to the ICU is not prohibitively high, and admission to the ICU should be considered for selected patients (registered with PROSPERO, CRD42020207209).

## INTRODUCTION

Since December 2019, the world has been gripped by COVID-19, the disease caused by severe acute respiratory syndrome coronavirus 2, with more than 174 million cases and 3.75 million deaths. The spectrum of COVID-19 spans from asymptomatic through moderate to severe. About 5% of all patients and 20% of hospitalized patients with COVID-19 may experience severe manifestations necessitating intensive care unit (ICU) admission.^1^ Mortality of patients with COVID-19 admitted in the ICU is high. In one meta-analysis,^2^ 31% of patients admitted to the ICU died, whereas in another, mortality ranged from 0% to 84.6%, with a pooled mortality of 41.6%.^3^

**CONTEXT**

**Key Objective**
Patients with cancer are at increased risk of complications from COVID-19 and may require admission to an intensive care unit (ICU). Although several studies have reported on outcomes of cancer patients with COVID-19, our systematic review and meta-analysis specifically investigated the mortality of cancer patients with COVID-19 admitted to the ICU (28 studies) or those with severe COVID-19 (four studies). This may help inform decisions whether to admit critically ill patients with cancer and COVID-19 to an ICU.
**Knowledge Generated**
The pooled mortality in cancer patients with COVID-19 admitted to an ICU was 60.2%; in cancer patients with severe COVID-19, pooled mortality was 59.4%.
**Relevance**
Mortality of critically ill cancer patients with COVID-19 is not prohibitively high. Decisions on ICU admission for these patients must be individualized taking into account the performance status of the patient and the potential for cure or significant palliation of the cancer.

Patients with cancer may be at increased risk of complications and mortality from COVID-19 owing to the systemic effects of malignancy; immune suppression after chemotherapy; treatment-related cardiovascular, renal, and pulmonary toxicities^4^; as well as the coexistence of comorbidities. Active cancer is associated with increased odds of death among patients with COVID-19.^5^ In two large series of cancer patients with COVID-19, mortality ranged from 13% to 28%.^6,7^ Some studies have found that patients with cancer had a higher risk of severe events and in-hospital mortality.^7-9^

Cancer patients with COVID-19 may develop serious complications necessitating ICU admission. In the setting of a global pandemic, allocation of intensive care resources may require triaging or prioritization of ICU admissions on the basis of outcomes in specific patient populations, such as those with COVID-19 and cancer.

An estimate of the mortality rate in cancer patients with COVID-19 admitted to the ICU on the basis of the available data could help in the planning and prioritization of patients for ICU admission. Although there are data to suggest that all-cause mortality and the need for ICU admission were higher in COVID-19 patients with cancer than those without cancer,^7-10^ other studies have found no difference between COVID-19 patients with and without cancer with respect to a composite outcome including death, intubation, or ICU admission.^11^ Very few studies have specifically reported the mortality of patients with cancer admitted in ICUs, and there are scarce data to aid in selection of critically ill cancer patients with COVID-19 for admission to the ICU or help in prognostication of outcome. Hence, we performed a systematic review and meta-analysis of the available literature to estimate the mortality among cancer patients with COVID-19 admitted to the ICU or those with severe COVID-19.

## METHODS

The review was prospectively registered on PROSPERO (CRD42020207209) and conducted according to Preferred Reporting Items for Systematic Reviews and Meta-Analyses guidelines.^12^ Ethics committee approval was not required.

### Data Sources

We searched for terms related to cancer, COVID-19, and intensive care. Exact search terms are in the Appendix Table A1. Various databases including PUBMED, MEDLINE, SCOPUS, and Web of Science were searched, supplemented by manually searching Cochrane Library and Google Scholar. All articles published from the first report of COVID-19 to October 31, 2020, were eligible to be included in the review.

### Study Selection

All studies in English including retrospective and prospective cohort studies, case-control studies, and case series were included if they reported adult patients (age ≥ 18 years) with cancer and COVID-19 who were admitted to the ICU. Where ICU admissions were not specified, patients with cancer and severe or critical COVID-19 were included and their outcomes were analyzed separately. Severe disease included clinical signs of pneumonia plus one of the following: respiratory rate > 30 breaths/min, severe respiratory distress, or SpO_2_ < 90% on room air. Critical disease included development of the acute respiratory distress syndrome, sepsis, or septic shock.^13^

In studies that included data on both cancer patients with COVID-19 admitted to the ICU and those with severe COVID-19, only the data for patients admitted to the ICU were extracted. Studies were excluded if the primary outcome was not reported or it was not possible to extract the outcome of cancer patients with COVID-19 from the publication. Preclinical studies, epidemiologic studies, descriptive studies, and randomized controlled trials or studies without a report on mortality outcomes in adult patients with cancer that were admitted to the ICU or had severe COVID-19 were excluded. Studies were imported to Rayyan—a Web and mobile app for systematic reviews—and independently screened by two reviewers^14^ (A.R.N. and S.C.V.). Disagreements were resolved through mutual discussion, and persistent disagreements were resolved by a third reviewer (J.V.D.). All three literature searchers were clinicians working with critically ill patients. After screening the title and abstracts, full-text studies were identified and were independently assessed by the two primary reviewers.

### Data Extraction and Quality Assessment

Data extraction and risk of bias assessment was performed on Microsoft Excel independently by two reviewers (S.C.V. and A.R.N.), with 20% of studies overlapped to assess reliability.

The extracted data points included study setting and design and stratification of patients with cancer on the basis of the severity of disease as defined by WHO criteria.^13,15^ The primary outcome was all-cause mortality in all patients. We also determined the primary outcome in the following subgroups: geographical location, cancer subtypes, presence of recent anticancer therapy (defined as therapy given within 1 month of diagnosis of COVID-19), patients receiving mechanical ventilation in the ICU, and presence of one or more comorbidities. Given the paucity of data and the variable length of follow-up in the included studies, we decided to include mortality regardless of the period of follow-up.

Secondary outcomes included the need for advanced support therapies in patients in the ICU and complications in patients with severe COVID-19. Where available, mortality data in noncancer COVID-19 patients admitted in the ICU from the same cohort were used to determine the odds of death for cancer patients compared with noncancer patients. The risk of bias assessment was carried out using a Modified Newcastle-Ottawa Scale,^16^ which reports three points for selection, two for comparability, and three for outcomes (Appendix Table A2) Funnel plot asymmetry generated using Public Health England tool was used to identify publication bias.^17^

### Data Synthesis

Meta-analysis was conducted using Open Meta-Analyst (CEBM, Brown University, Providence, RI).^18^ The pooling of the results was performed using the Der Simonian-Laird random-effects model. Summary of findings tables were constructed using GRADE pro GDT (GRADEpro Guideline Development Tool [Software], McMaster University, 2020 [developed by Evidence Prime Inc]).^19^ The primary outcome identified for meta-analysis was the pooled mortality rate in patients with COVID-19 admitted to the ICU or cancer patients with severe COVID-19. Subgroup analysis for the primary outcome was performed after grouping of study patients by geographical location (Europe, United States, China, and multinational), hematologic versus other cancers, the use of recent anticancer therapy versus former anticancer therapy, sample size (≤ 25 *v* > 25 patients), and presence of comorbidities. Patients requiring invasive mechanical ventilation were analyzed separately for pooled mortality outcomes. The odds of mortality in COVID-19 patients with cancer versus COVID-19 patients without cancer among patients admitted to the ICU were also estimated. Primary and secondary outcomes were reported and graded using GRADEpro GDT,^19^ tabulated in the summary of findings tables. Meta-regressions for mortality were performed for number of days since December 2019.

## RESULTS

**FIG 1:**
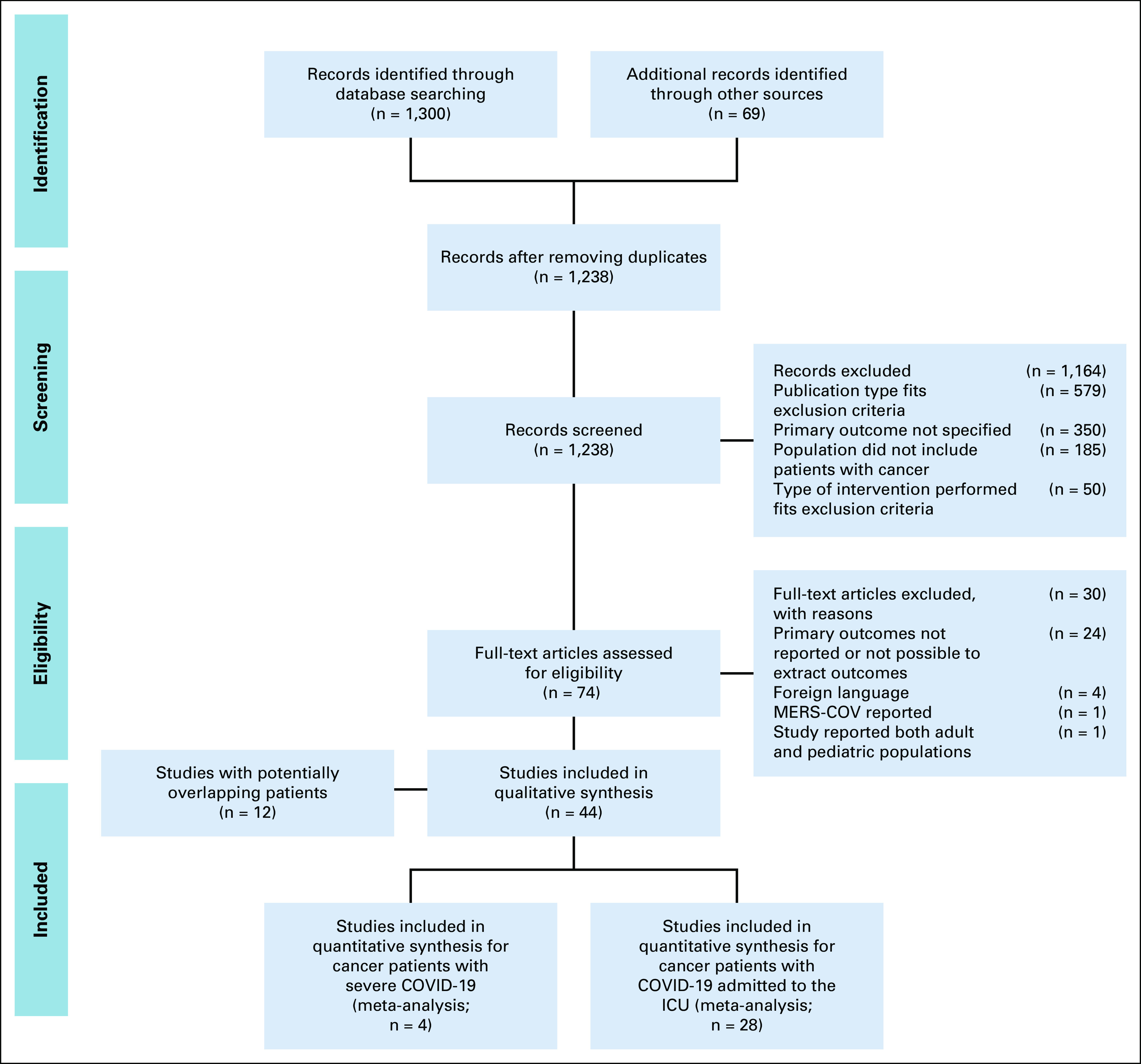
PRISMA chart listing included and excluded studies. ICU, intensive care unit; MERS-COV, Middle East respiratory syndrome coronavirus; PRISMA, Preferred Reporting Items for Systematic Reviews and Meta-Analyses.

**FIG 2:**
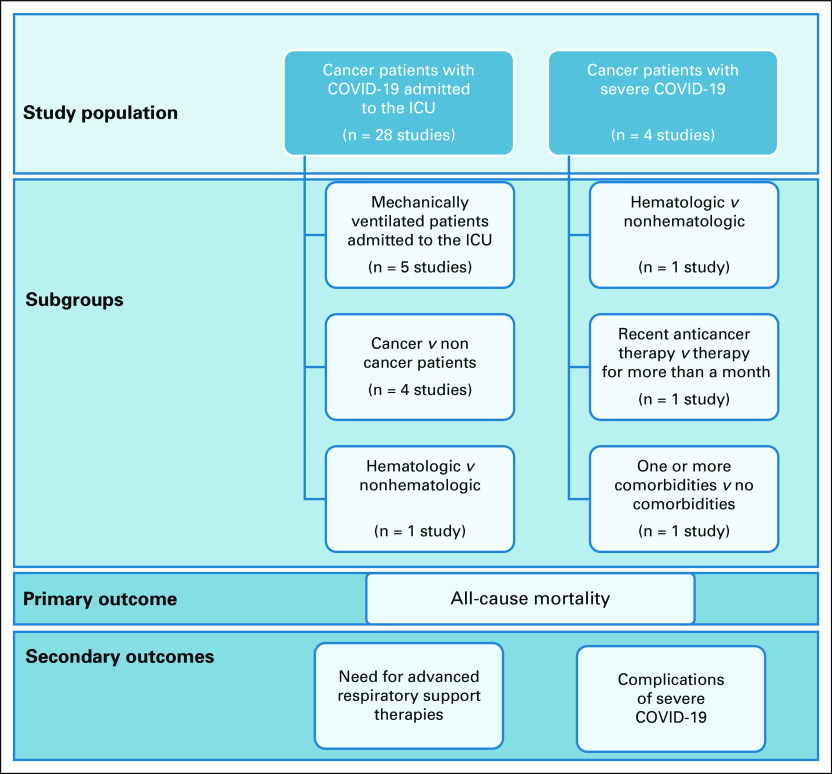
Schematic representation of primary and secondary outcomes analyzed. ICU, intensive care unit.

One thousand three hundred studies were identified on electronic literature search, with 69 studies identified on manual searching. After removing duplicates, 1,238 studies were screened by title and abstract; 74 full-text articles were identified for eligibility, of which 44 studies reporting the primary and other outcomes were included for data extraction. Reasons for exclusion can be identified in the Preferred Reporting Items for Systematic Reviews and Meta-Analyses chart (Fig 1). There were 13 studies from China with potentially overlapping patients.^20-32^ On the basis of overlapping study duration and hospital location, a decision was taken to include only the most recent study (Yang et al)^29^ in the meta-analysis for studies reporting mortality in cancer patients with COVID-19 admitted to the ICU and the largest cohort (Zhang et al)^25^ for studies reporting mortality in cancer patients with severe COVID-19 (Appendix Table A3). Furthermore, two studies detailing outcomes and risk factors for ICU patients with COVID-19 in Lombardy, Italy, were identified.^33,34^ A decision was taken to include the study with the later date of publication.^34^ Twenty-eight studies were included for meta-analysis; these included patients from Asia (three studies),^29,35,36^ the Americas (10 studies),^5,37-45^ Europe (13 studies),^9,34,46-56^ and multinational registries (two studies).^6,57^ The 28 studies that reported mortality in cancer patients with COVID-19 admitted to the ICU included a total of 1,276 patients, with dates of recruitment ranging from January 23 to June 11, 2020. Four studies that included 106 patients and reported mortality in cancer patients with severe COVID-19 were from China (one study),^25^ Spain (two studies),^58,59^ and United Kingdom (one study).^60^ Figure 2 summarizes the studies included and the subgroups studied with the primary and secondary outcomes. Five studies with a total of 131 patients reported mortality outcomes of mechanically ventilated cancer patients in the ICU.^42,43,51,53,56^ Two studies reporting mortality of patients with invasive mechanical ventilation were excluded from the analysis because ICU admission was not mentioned; the numbers including mechanically ventilated patients exceed the number of patients in the ICU cohort, suggesting that not all mechanically ventilated patients may have been admitted to the ICU.^38,45^ Of studies reporting mortality in cancer patients with COVID-19 admitted to the ICU, four studies reported mortality outcomes for noncancer patients with COVID-19^5,26,34,54^ and one study reported mortality outcomes on the basis of cancer subtype.^54^ For studies reporting outcomes of cancer patients with severe COVID-19, one study reported mortality outcomes on the basis of cancer subtype, recent anticancer therapy, or presence of one or more comorbidities.^6^ Details of studies included and outcomes are summarized in Table 1.

**TABLE 1:**
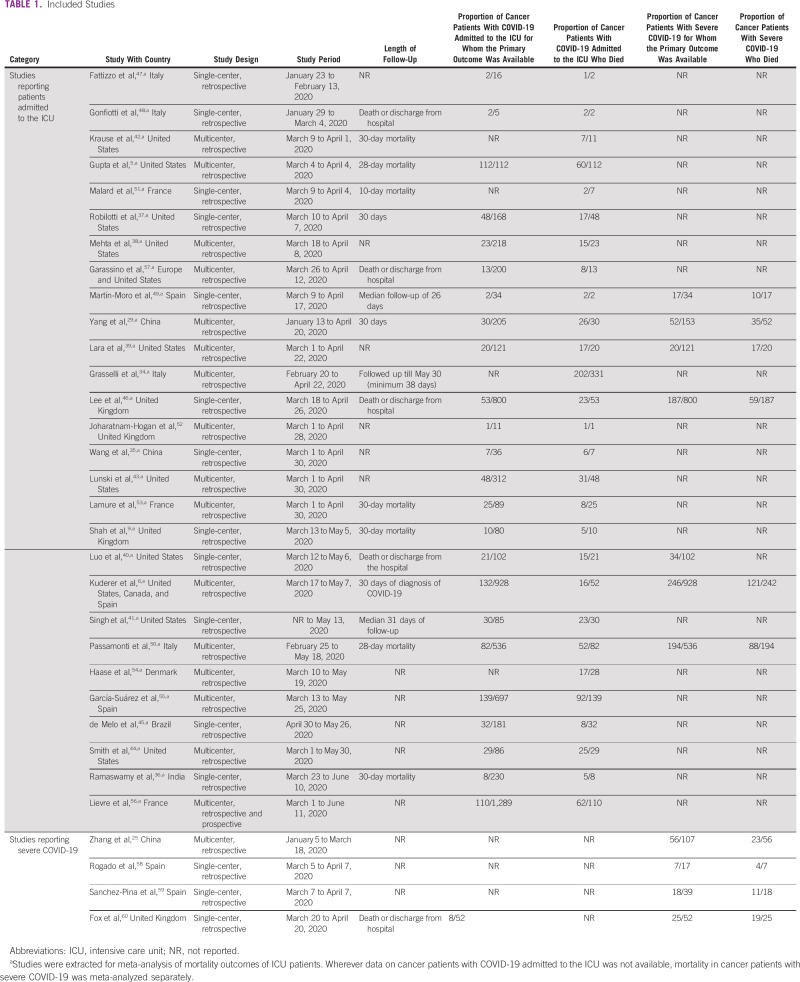
Included Studies

Mortality was variably reported in the included studies as death in ICU, in hospital, at 28 days, at 30 days, or on a cutoff date (Table 1). The risk of bias assessment on the basis of the Modified Newcastle-Ottawa Scale was a median 7/8 (Appendix Table A4).

### Cancer Patients With COVID-19 Admitted to the ICU

**FIG 3:**
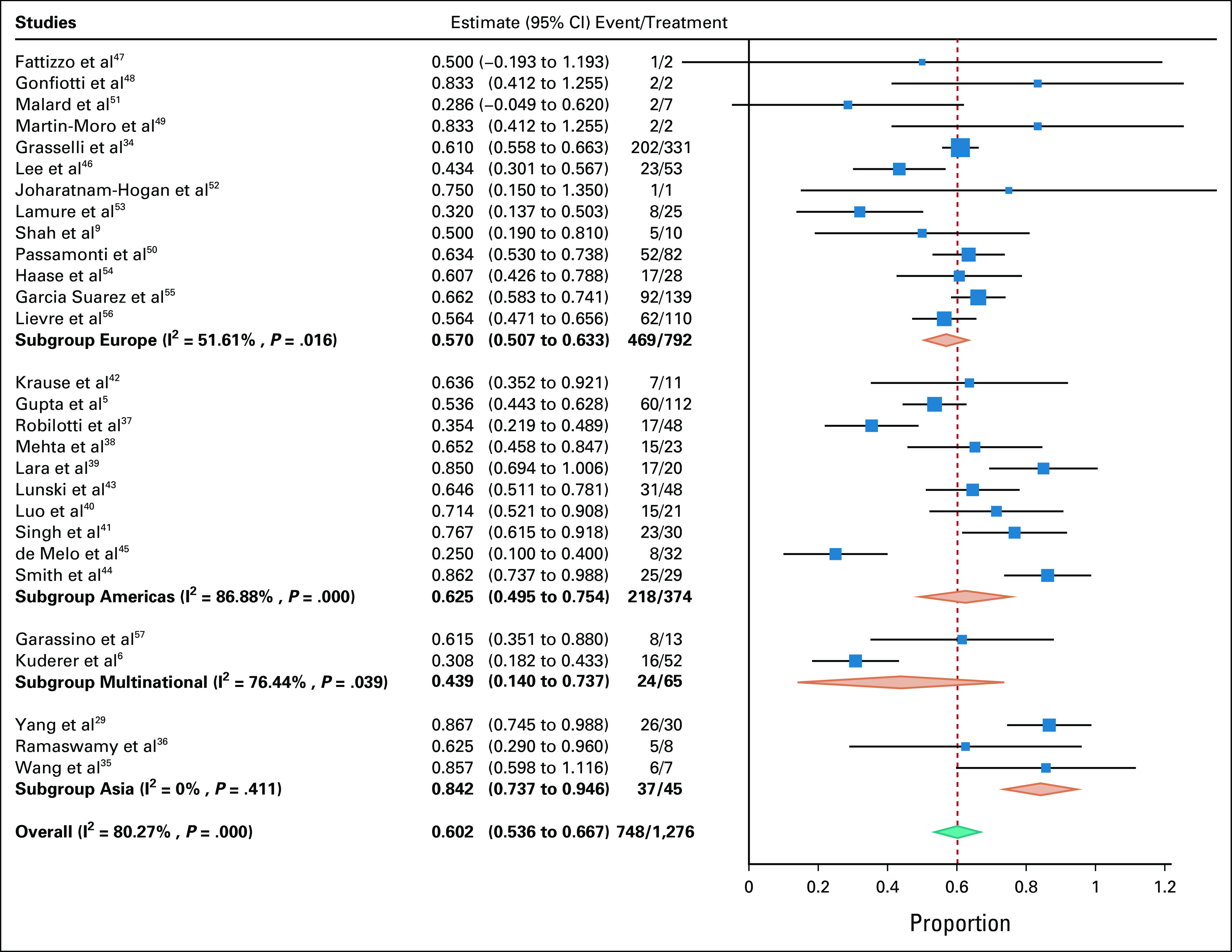
Meta-analysis, including subgroup analysis on geographical location.

For cancer patients with COVID-19 admitted to the ICU, the pooled mortality rate was 60.2% (95% CI, 53.6 to 66.7), with heterogeneity reported at I^2^ = 80.27% (Table 2, Fig 3). The largest study was that of Grasselli et al,^34^ with a cohort of 331 cancer patients with COVID-19 admitted to the ICU; however, a sensitivity analysis with the study excluded did not significantly affect mortality (60.2%; 95% CI, 52.8 to 67.6) or heterogeneity (I^2^ = 80.98%).

**TABLE 2:**
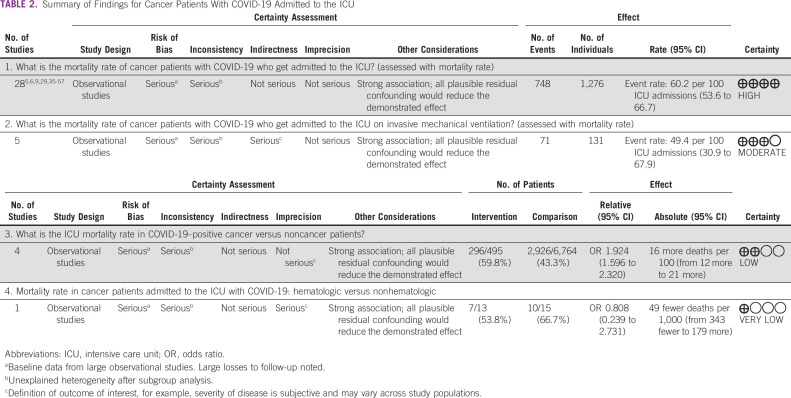
Summary of Findings for Cancer Patients With COVID-19 Admitted to the ICU

**FIG A1:**
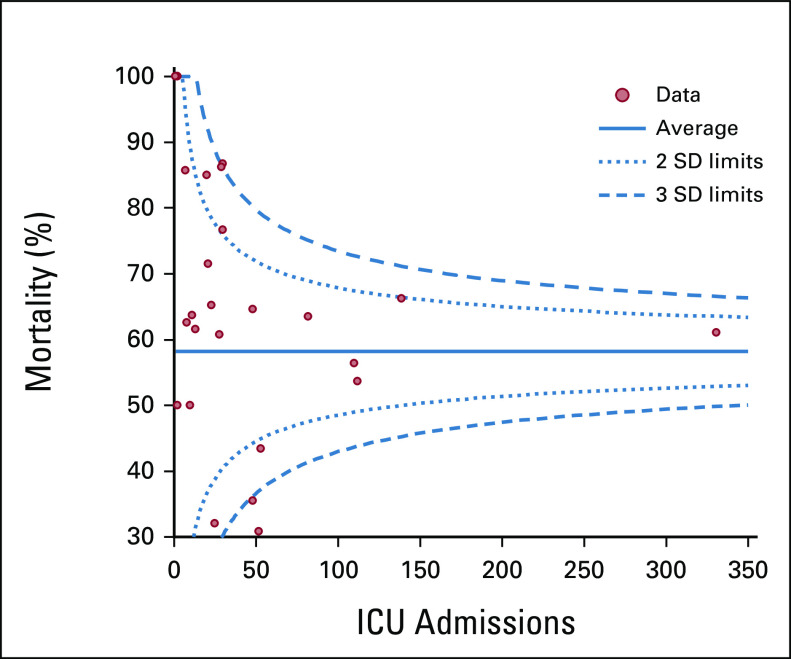
Funnel plot for assessing publication bias. Cancer patients with COVID-19 admitted to the ICU (*x*-axis) versus ICU mortality rate (*y*-axis); reports studies within and beyond ±3 SD of pooled proportion of ICU mortality. ICU, intensive care unit; SD, standard deviation.

Pooled mortality rate of patients with cancer on invasive mechanical ventilation in the ICU (five studies, 131 patients) was 49.4% (95% CI, 30.9 to 67.9; I^2^ = 78.1%). The mortality in cancer patients with COVID-19 admitted to the ICU was significantly higher than that in noncancer patients with COVID-19 admitted to the ICU (59.8% [95% CI, 54.8 to 64.8] *v* 42.3% [95% CI, 33.6 to 51.1]; odds ratio [OR] 1.924; 95% CI, 1.596 to 2.320). In one study,^54^ mortality of patients with hematologic malignancies as compared with nonhematologic malignancies did not differ significantly (53.8% [95% CI, 26.7 to 80.9] *v* 66.7% [95% CI, 42.8 to 90.5]; Table 3). Subgroup analysis on the basis of geographical location revealed significant reduction in heterogeneity and increased mortality for studies from Asia (84.2% [95% CI, 73.7 to 94.6], I^2^ = 0) and significant reduction in heterogeneity for Europe (57% [95% CI, 50.7 to 63.3], I^2^ = 51.61). Estimates for mortality by number of centers and sample size are detailed in Appendix Table A5. Funnel plot asymmetry was negative, with two reporting mortality < 3 standard deviations (Appendix Fig A1).

**TABLE 3:**
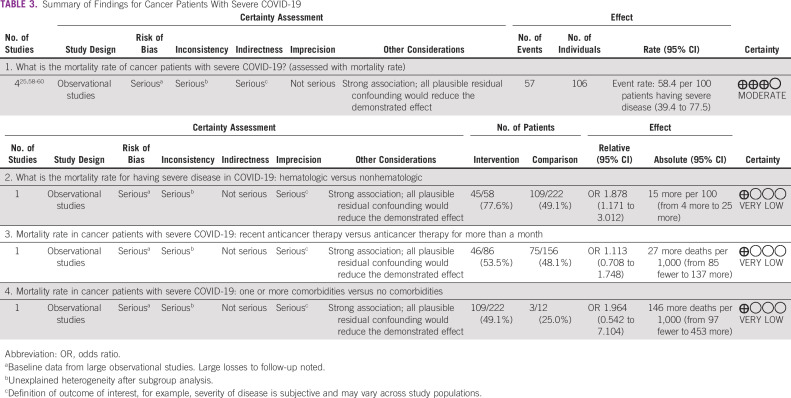
Summary of Findings for Cancer Patients With Severe COVID-19

Studies reporting percentage of ICU patients who required advanced respiratory support therapies can be found in Appendix Table A6.

**FIG A2:**
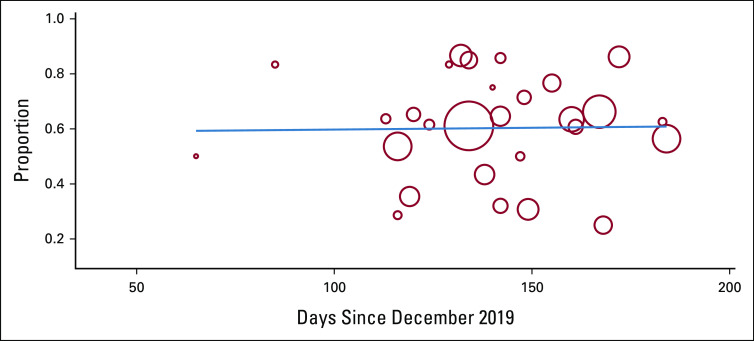
Meta-regression for proportion of patients who died in the intensive care unit (on the *x*-axis) versus date of recruitment. (on the *y*-axis). The circles indicate the proportion of patients dying in each study. The circle size is proportional to the precision of the estimate.

Meta-regressions for ICU mortality showed that mortality in cancer patients with COVID-19 admitted to the ICU did not differ on the basis of the date of recruitment (Appendix Fig A2).

### Cancer Patients With Severe COVID-19

**FIG A3:**
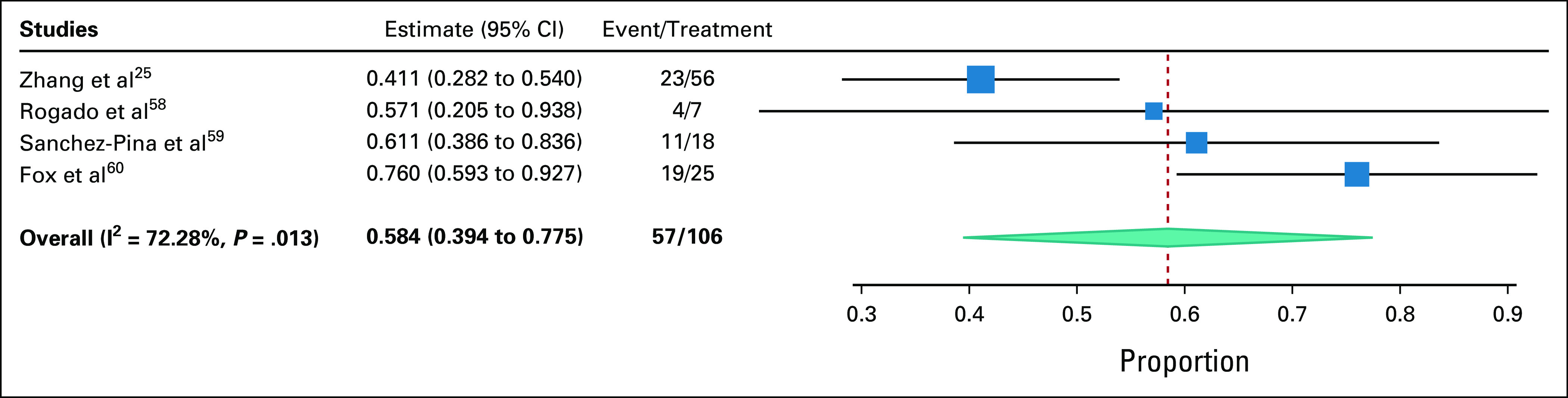
Meta-analysis of cancer patients with severe COVID-19.

The pooled mortality rate in cancer patients with severe COVID-19 was 58.4% (95% CI, 39.4 to 77.5) with I^2^ of 72.28% (Appendix Table A5, Appendix Fig A3). Along with mortality in cancer patients with COVID-19 admitted to the ICU, mortality in cancer patients with severe COVID-19 is reported in Table 2. One study of 246 patients with severe COVID-19 reported higher mortality among patients with hematologic versus those with nonhematologic cancers (77.6% [95% CI, 68.9 to 88.3] *v* 41.3% [95% CI, 38.2 to 41.4]; OR 1.878; 95% CI, 1.171 to 3.012; Appendix Table A7), but there was no difference among patients with comorbidity compared with those without any comorbidity (49.1% [95% CI, 42.5 to 55.7] *v* 25% [95% CI, 5 to 49.5]; OR 1.964; 95% CI, 0.542 to 7.104) or in patients who had received recent anticancer therapy compared with those who had not received such therapy (53.5% [95% CI, 42.9 to 64] *v* 48.1% [95% CI, 40.2 to 55.9]; OR 1.113; 95% CI, 0.708 to 1.748^6^; Appendix Table A7). Our analysis reports the prevalence of pulmonary complications (49.7%), cardiac complications (14.3%), sepsis (11.5%), and renal complications (8.7%; Appendix Table A8).

## DISCUSSION

Our analysis suggests that the mortality in cancer patients with COVID-19 who are admitted to ICUs or who have severe COVID-19 is nearly 60%. Among cancer patients with severe COVID-19, the odds of death were higher in patients with hematologic cancers. We also found that cancer patients with COVID-19 in the ICU had a two-fold increase in odds of death compared with COVID-19 patients without cancer.

Patients with cancer may be immunosuppressed because of disease or treatment and have cancer- or treatment-related organ dysfunction. Studies comparing cohorts of cancer and noncancer patients have found that patients with hematologic malignancies have a higher mortality rate and incidence of ICU admissions.^9,54^ Another small study found no significant difference in terms of overall survival between solid-tumor and hematologic patients, although patients with a hematologic malignancy showed a nonsignificant trend for earlier occurrence of severe events compared with solid-tumor patients.^61^ Among cancer patients with COVID-19, cancer type^6^ and active treatment with chemotherapy, immunotherapy, targeted therapies, hormonal therapy, surgery, or radiotherapy within four weeks of diagnosis were not associated with increased adverse outcomes.^46^ Unlike these studies that looked at all patients with cancer and COVID-19, our study was focused on cancer patients with COVID-19 admitted to the ICU or those with severe disease. Our analysis too did not reveal a statistically significant difference in mortality in patients with recent anticancer therapy and presence of one or more comorbidities possibly because of paucity in available data.

Before the pandemic, the unadjusted pooled mortality of critically ill cancer patients in studies published between 2005 and 2015 was 47.1%.^62^ With advances in oncology and intensive care, mortality rates of critically ill cancer patients have further improved and are below 30% in the ICU and below 40% in hospital.^63^ On the basis of such outcomes, most authorities recommend that patients with cancer (with the exception of those with very poor performance status and advanced disease for whom no therapeutic options are available) should be admitted to the ICU for aggressive treatment or a therapeutic ICU trial for about 5 days.^63^ During the COVID-19 pandemic, a meta-analysis by Armstrong et al^3^ found that the mortality of critically ill patients with COVID-19 has decreased over the course of the pandemic from more than 50% to 40%. The mortality rate of 60% in cancer patients with COVID-19 admitted to the ICU suggests that outcomes in cancer patients with COVID-19 are not prohibitively high. Furthermore, a majority of patients in the included studies were treated before the availability of the results of trials that suggested that dexamethasone and tocilizumab may be beneficial in patients who receive either oxygen or mechanical ventilation.^64-66^ On the basis of such recent advances in care of patients with COVID-19, our meta-analysis may overestimate the true mortality of critically ill cancer patients.

Our meta-regression falls short of showing a statistically significant difference in mortality over the period analyzed, again because of the small numbers of patients studied.

The decision regarding the admission of patients with cancer to the ICU in the setting of the COVID-19 pandemic is dictated by various locoregional factors such as availability of ICU resources, institutional policies, underlying cancer diagnosis, a decision not to escalate to ICU for futility in patients with advanced-stage cancer and end-of-life care decisions during ICU care. In a cohort of 928 patients, patients with progressive cancer died at a numerically higher rate without ICU admission than those who were admitted to an ICU.^6^ This suggests that aggressive interventions might have already been restricted in these subpopulations and partially explains the similar mortalities in cancer patients with COVID-19 admitted to the ICU and those classified as severely ill. Studies included in our systematic review have noted refusal of ICU admission or limitation of beds.^58,59^ In one multicenter study, only 10% of eligible patients were admitted to the ICU.^57^ Similarly, in another study, patients admitted in the ICU were younger and had a lower Charlson Comorbidity Index,^67^ suggesting a selection bias. Hantel et al reported local crisis standards of care in the United States that deprioritize patients with cancer in favor of less aggressive interventions, often without sufficient precision to differentiate different survival patterns of cancer subtypes.^68^ Considerations such as these may have resulted in several patients being denied admission to ICUs, explaining the paucity of and lack of granularity in the data.

As the pandemic wanes in several parts of the world, there will be occasions when patients with cancer will require admission to the ICU. We believe that on the basis of the results of our meta-analysis, mortality in critically ill patients with COVID-19 and cancer is not prohibitively high, and patients with COVID-19 must not be denied ICU admission only on the grounds that they have cancer. These decisions will need to be individualized taking into account the performance status of the patient and the potential for cure or significant palliation of the cancer.

To our knowledge, this is the first systematic review exploring mortality in cancer patients with COVID-19 admitted to the ICU, or with severe COVID-19.^13,15^ Furthermore, the median risk of bias for our review is 7/8, and the grade of evidence for the primary outcome using the GRADEpro was moderate to high and there is a low risk of publication bias.

Patients were included in meta-analysis on the basis of data that were available for extraction from studies in English that were published or accepted for publication. We did not contact authors for individual patient data. Most studies were performed before publication of trials of therapies such as dexamethasone and tocilizumab. Our review is based on observational studies, and the high heterogeneity noted across studies suggests that results of this review need to be interpreted with caution. The severity of illness in terms of physiologic parameters such as the Acute Physiology and Chronic Health Evaluation score or the Sequential Organ Failure Assessment score is not available.^69,70^ Mortality data are also limited by the numbers of patients still in ICU or hospital on the cutoff date for estimation of mortality. Furthermore, despite the inclusion of 28 studies, we did not have sufficient granularity in the data to definitively determine differences in outcomes in important subgroups, such as hematologic versus solid-tumor malignances and patients receiving active chemotherapy versus those not receiving chemotherapy.

In summary, the results of our meta-analysis suggest that cancer patients with COVID-19 who require admission to an ICU or those who have severe COVID-19 experience high mortality. However, denying ICU admission to patients with COVID-19 only because they have cancer may not be justified. Targeted interventions to prevent transmission of severe acute respiratory syndrome coronavirus 2 among patients with cancer and early therapeutic interventions in those with COVID-19 are likely to remain very important in the near future.

## Figures and Tables

**TABLE A1:**
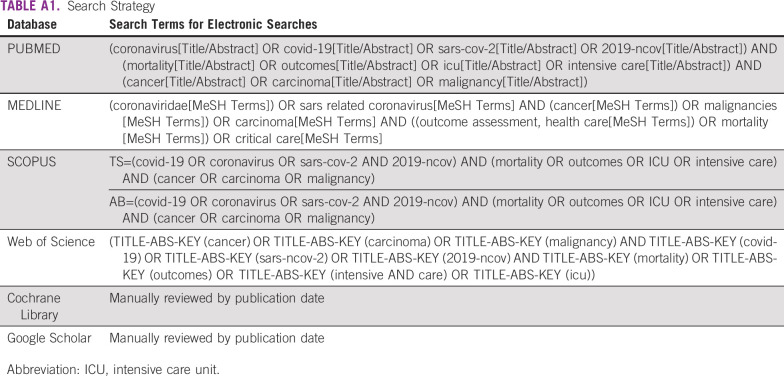
Search Strategy

**TABLE A2:**
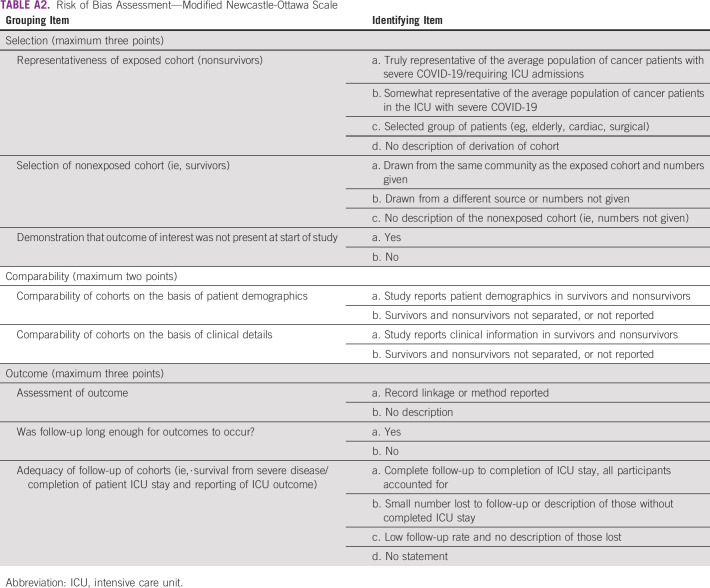
Risk of Bias Assessment—Modified Newcastle-Ottawa Scale

**TABLE A3:**
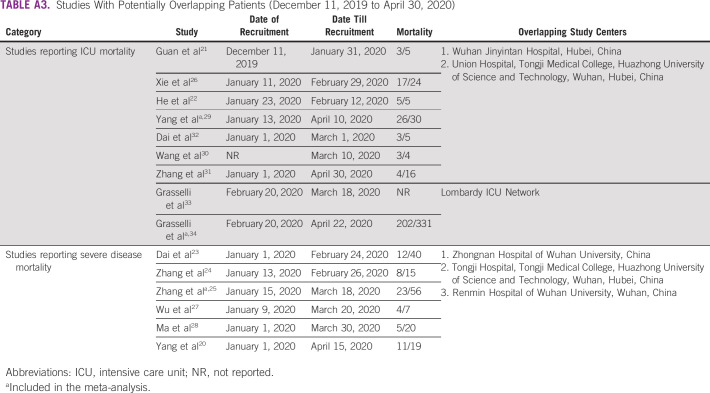
Studies With Potentially Overlapping Patients (December 11, 2019 to April 30, 2020)

**TABLE A4:**
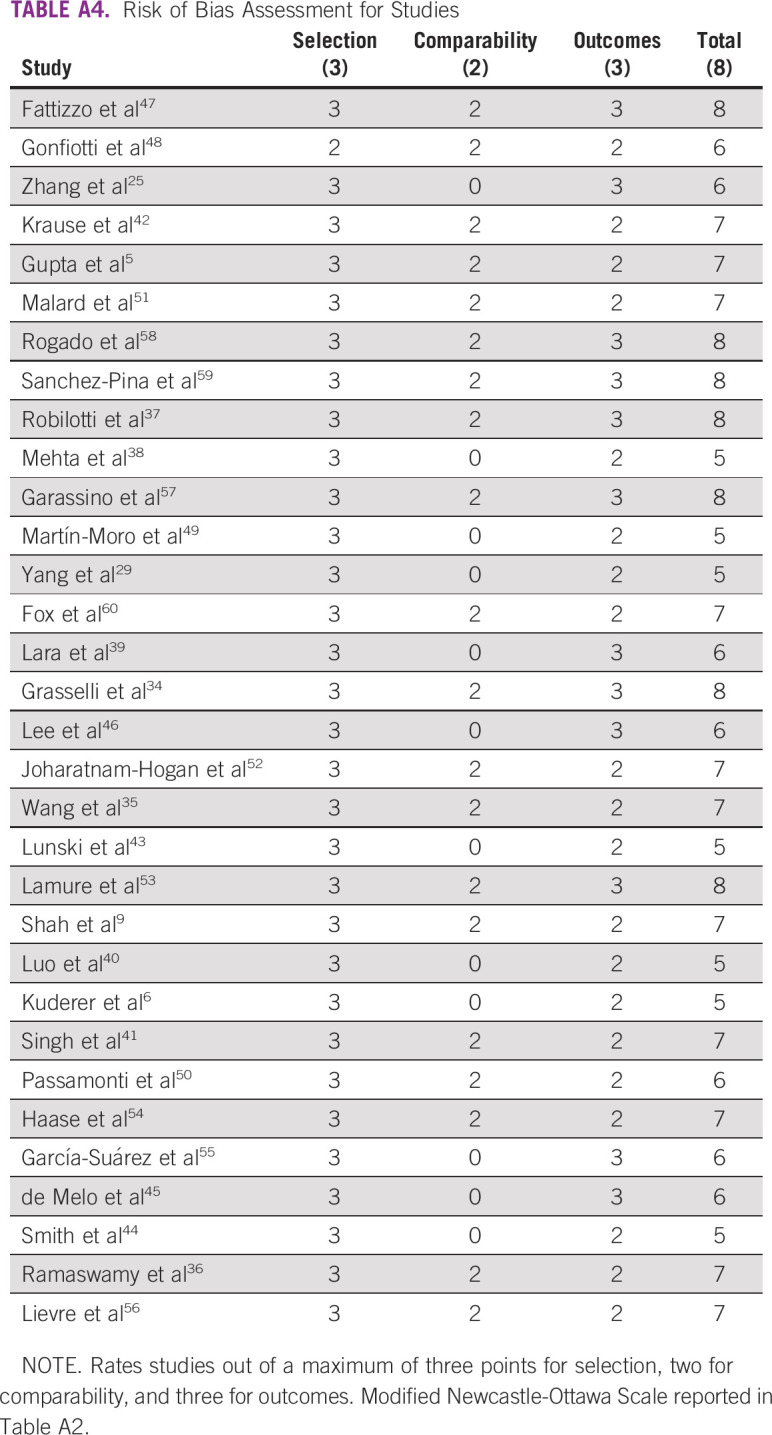
Risk of Bias Assessment for Studies

**TABLE A5:**
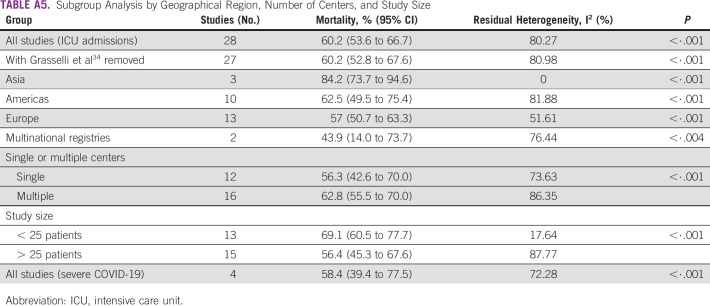
Subgroup Analysis by Geographical Region, Number of Centers, and Study Size

**TABLE A6:**
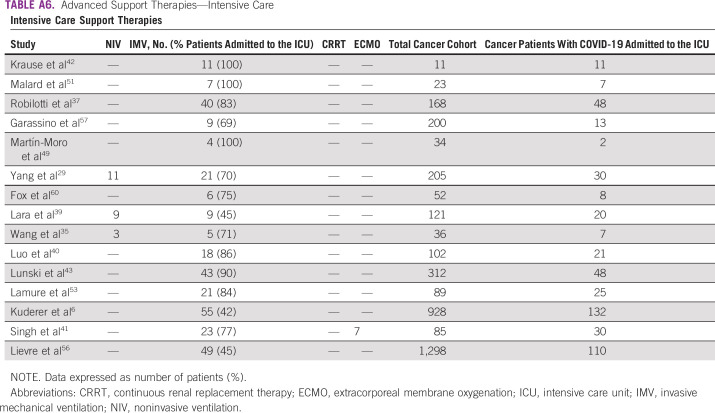
Advanced Support Therapies—Intensive Care

**TABLE A7:**
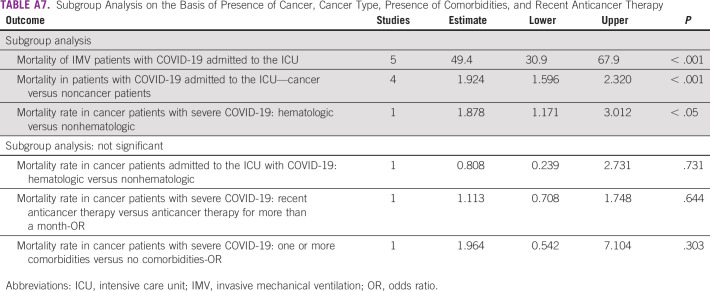
Subgroup Analysis on the Basis of Presence of Cancer, Cancer Type, Presence of Comorbidities, and Recent Anticancer Therapy

**TABLE A8:**
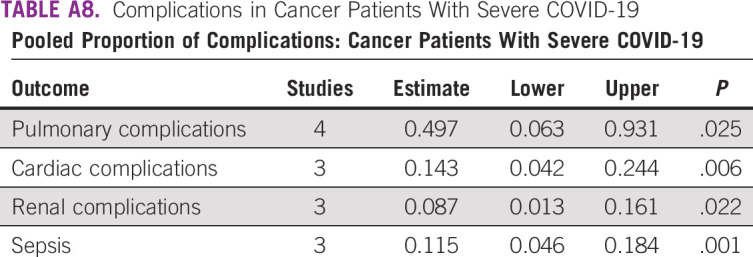
Complications in Cancer Patients With Severe COVID-19
